# Oxidative and Molecular–Structural Alterations of Spermatozoa in Swine and Ram Exposed to the Triazole Ipconazole

**DOI:** 10.3390/toxics13030176

**Published:** 2025-02-28

**Authors:** Cristian Falero, Wilfredo Huanca, Luis Barrios-Arpi, Boris Lira-Mejía, Olger Ramos-Coaguila, Edith Torres, Eva Ramos, Alejandro Romero, Mariella Ramos-Gonzalez

**Affiliations:** 1 Zootecnia an Animal Production Laboratory, Faculty of Veterinary Medicine, Major National University of San Marcos, Lima 15021, Peru; nilton.falero@unmsm.edu.pe (C.F.); olger.ramos@unmsm.edu.pe (O.R.-C.); 2 Reproduction Laboratory, Faculty of Veterinary Medicine, Major National University of San Marcos, Lima 15021, Peru; whuancal@unmsm.edu.pe; 3 Animal Physiology Laboratory, Faculty of Veterinary Medicine, Major National University of San Marcos, Lima 15021, Peru; lbarriosa@unmsm.edu.pe (L.B.-A.); bliram@unmsm.edu.pe (B.L.-M.); 4 Reproduction Laboratory, School of Veterinary and Zootecnic Medicine, Jorge Basadre Grohmann University, Tacna 23001, Peru; etorresh@unjbg.edu.pe; 5 Department of Pharmacology and Toxicology, Faculty of Veterinary, Complutense University of Madrid, 28040 Madrid, Spain; eva.ramos@ucm.es (E.R.); manarome@ucm.es (A.R.)

**Keywords:** spermiotoxicity, ipconazole, oxidative stress, structural biomarkers

## Abstract

Triazole pesticides are widely used throughout the world, but their abuse causes toxic effects in non-targeted organisms. In the present study, the cytotoxic effect of the triazole ipconazole was evaluated in porcine and ram spermatozoa. Ipconazole significantly reduced sperm viability, increased ROS levels, altered catalase and SOD enzyme activity, and caused alterations in the molecular mRNA expression of structural biomarkers (PRM1, ODF2, AKAP4, THEG, SPACA3 and CLGN) related to fertility in males, as well as the overexpression of BAX (cell death) and ROMO1 (oxidative stress) mRNA. Our results indicate that the fungicide triazole is involved in cellular, enzymatic and molecular alteration of porcine and ram spermatozoa, and is possibly a factor in the development of infertility in male mammals.

## 1. Introduction

Industrial pesticides are synthetic chemical compounds used alone or in mixtures to prevent or eradicate pests (weeds, fungi, insects, etc.), affecting agricultural fields, including vectors of human and animal diseases. In addition, pesticides help improve agroeconomic performance [1]. Despite their advantages in animal production and the reduction in crop diseases, pesticides are considered environmental pollutants and toxic to the public and animal health as they remain in the atmosphere and subsequently contaminate water, food and soil. Several key structural factors influence how long a pesticide remains active before breaking down. One of the most significant factors is the presence and type of functional groups. Halogen substituents (e.g., chlorine, fluorine) generally increase persistence by making molecules more resistant to microbial degradation and hydrolysis. In contrast, ester bonds are highly susceptible to hydrolysis, leading to a faster breakdown. Similarly, phosphorus-containing bonds (P–O, P–S) in organophosphate pesticides can be readily oxidized, reducing their persistence and influencing their environmental fate [2]. It is important to note that the specific health effects of pesticide exposure can vary depending on the type of pesticide, the level and duration of exposure, and individual susceptibility factors. Long-term exposure has been associated with an increased risk of neurological disorders, mutagenicity, carcinogenicity, teratogenicity and endocrine disruption, which depend on the type of pesticide, exposure dose, exposure time and its solubility [3]. However, establishing a definitive link between chronic health conditions and pesticide exposure can be challenging due to the delayed onset of symptoms, and the presence of multiple environmental and genetic contributing factors [4].

Chemicals used in agriculture are often considered relevant factors in developing physiological alterations in the reproductive system. Reproductive alterations produced by agrochemicals can be endocrine disruptors or gonadal [5]. Agrochemicals such as pesticides can alter seminal parameters, with total sperm count, motility and morphology being the most frequently worsened [6].

Sperm concentration is the most frequently measured semen parameter in the presence of pesticide exposure. There is a strong association between decreased sperm concentration and the presence of pyrethroid [7], organophosphate [8], BPA [9] and phthalate [10] pesticide metabolites in urine. Instead, seminal volume and total sperm count are parameters that may or may not be altered by the effect of pesticides. Synthetic pyrethroids, OPs and phthalates have also been found to cause sperm aneuploidy and an altered X:Y ratio [7,8,11].

Triazole fungicides have been widely used in agriculture worldwide, and their misuse and abuse have been shown to be responsible for producing physiological alterations in non-target organisms. For example, continuous exposure to penconazole reduces testosterone levels, causes spermatogenic alterations, and impairs Sertoli and Leydig cell morphology [12]. According to previous studies, tebuconazole caused a decrease in the number of germ cells and increased oxidative stress-related genes in fetal mouse testis [13]. Triazole compounds also affected chick testicular functions by reducing cell viability, steroidogenesis and lactate production. Furthermore, the exposure of spermatozoa to triazoles causes a decrease in sperm motility, and an increase in sperm abnormalities and ROS production [14]. For this reason, the present study aimed to evaluate the oxidative and structural physiological alterations of porcine and ram spermatozoa due to the fungicide ipconazole.

## 2. Materials and Methods

### 2.1. Chemicals

Ipconazole was obtained from LGC Standards (Middlesex, TW11 0LY, UK). Gentamicin, streptomycin and penicillin G; 2′,7′-dichlorofluorescin-diacetate (DCFH-DA); Dulbecco’s phosphate buffered saline and dimethyl sulfoxide were obtained from Sigma-Aldrich (Saint Louis, MO, USA). HyClone Dulbecco’s modified eagle medium (DMEM)/F12 was obtained from Cytiva (Marlborough, MA, USA). The catalase and superoxide dismutase (SOD) activity assay kit was obtained from Invitrogen (Thermo Fisher Scientific, Waltham, MA, USA). A commercial kit from MACHEREY-NAGEL (Düren, Germany) was used for the RNA extraction assay; a commercial kit from PCR Biosystems (London, UK) was used for cDNA synthesis; and the MasterMix qPCR ICGreen was obtained from NIPPON GENETICS (Düren, Germany). All other chemicals were of the highest grade available.

### 2.2. Sperm Samples and Treatment

Semen samples from swine and ram were donated by the Reproductive Biotechnology Laboratory of the Universidad Nacional Mayor de San Marcos. Semen was cryopreserved in straws in liquid nitrogen at −196 °C. The straws were slowly thawed in a heat bath for 1 min at 37 °C, and then the sperm were incubated for 2 h in culture medium at 37 °C and 5% CO_2_. Different concentrations of sperm were used depending on the assays performed (sperm viability, ROS production, SOD activity, catalase activity and qPCR assay). The sperm were exposed to ipconazole concentrations of 1, 5, 10, 50 and 100 µM, and to a control without ipconazole. All assays were performed in triplicate.

### 2.3. Sperm Viability

For sperm viability, 10^6^ porcine and ram spermatozoa were seeded in 200 µL of DMEM-F12 in 1.5 mL cryovials for 2 h with ipconazole at 37 °C and 5% CO_2_, in triplicate. After treatment with ipconazole, sperm viability was counted using an optical microscope and a Neubauer chamber [15]. Then, 10 µL of sample was placed on a pre-warmed slide at 37 °C, the coverslip was attached and motile spermatozoa were evaluated in different fields of the slide with a phase contrast microscope at 400× magnification. Approximately 200 spermatozoa in at least 5 fields per replicate (n = 3) were evaluated to determine the percentage of different motile categories (total and progressive). Calculations were performed to determine total motility and progressive motility.

Total motility is the total number of motile spermatozoa observed/total number of spermatozoa counted.

### 2.4. Reactive Oxygen Species (ROS) Generation

Next, 10^6^ porcine or ram spermatozoa were cultured in a phenol red-free culture medium in black 96-well plates at 37 °C and 5% CO_2_, and ipconazole treatments were immediately added in triplicate. After 1 ½ h of incubation, DCFH-DA (10 µM, dissolved in DMSO) was added and after 2 h of incubation, ROS production was recorded on a FLX800 fluorometer (BioTek, Winooski, VT, USA) with an excitation of 485 nm/emission 528 nm wavelength. Data were normalized as percentages with respect to the control (100%) [16,17].

### 2.5. Catalase Activity Assay

For this assay, the catalase colorimetric activity assay kit (Invitrogen, Thermo Fisher Scientific, MA, USA) was used according to the manufacturer’s guidelines. Briefly, 10^7^ cells were homogenized with the kit-supplemented buffer at 4 °C. Then, the homogenate was centrifuged at 10,000× *g*/15 min/4 °C, and the supernatant was immediately processed. In a 96-well plate, 25 µL of the sample was mixed with 25 µL of hydrogen peroxide reagent for 30 min at room temperature. Then, 25 µL of horseradish peroxidase was added and incubated for 15 min at room temperature. Spectrophotometric reading was performed at 560 nm (Agilent Technologies, Santa Clara, CA, USA), and optical density values were calculated from the standard curve fit to obtain catalase activity in U/mL/mg tissue. Data were normalized as percentages with respect to the control (100%) [16].

### 2.6. Superoxide Dismutase (SOD) Activity Assay

For this assay, the SOD colorimetric activity assay kit (Invitrogen, Thermo Fisher Scientific, MA, USA) was used according to the manufacturer’s guidelines. Briefly, 10^7^ cells were homogenized with the kit-supplemented phosphate-buffered saline at 4 °C. Then, the homogenate was centrifuged at 1500× *g*/10 min/4 °C and the supernatant was immediately processed. In a 96-well plate, 10 µL of the sample was mixed with 50 µL of 1X Substrate, and 25 µL of 1X Xanthine Oxidase into each well was added and incubated for 20 min at room temperature. The spectrophotometric reading was performed at 450 nm (Agilent Technologies, CA, USA), and optical density values were calculated from the standard curve fit to obtain SOD activity in U/mL. Data were normalized as percentages with respect to the control (100%) [18].

### 2.7. qPCR Analysis

For qPCR analysis, 10^7^ cells were incubated with the ipconazole treatments for 2 h in 1.5 mL tubes. After, the tubes were centrifuged at 12,000× *g*/10 min/4 °C and the supernatant was removed. Total RNA extraction was performed according to the NucleoSpin-RNA-Plus kit manufacturer’s instructions (MACHEREY-NAGEL, Germany), and concentrations were determined using a nanospectrophotometer (NanoDrop Lite Plus, Thermo Fisher Scientific, MA, USA) obtaining A260/A280 ratio values between 1.9 and 2.1. Retrotranscription was performed according to the specifications of the cDNA synthesis kit (PCRBiosystems, Wayne, PA, USA). For real-time PCR, MasterMix ICgreen (Nippon Genetics, Duren, Germany) was used according to the manufacturer’s specifications. The thermocycling protocol was 95 °C/2 min, 40 cycles of 5 s/95 °C and 30 s/60 °C in a Bio-Rad CFX (BioRad, Hercules, CA, USA). The primers used (Supplement Figure S1) were synthesized by Sigma-Aldrich (Saint Louis, MO, USA) [14,15].

### 2.8. Statistical Analysis

Data were analyzed using GraphPad Prism 8 statistical software. The results are presented as a percentage (%) or fold change compared to the control, and are expressed as the mean ± SEM per group. Significant differences between the control and treated groups were determined using a one-way ANOVA followed by Tukey’s post hoc test. The results were considered significant at * *p* < 0.05, ** *p* < 0.01 or *** *p* < 0.001.

## 3. Results

This study evaluated the oxidative capacity and structural alterations in porcine and ram spermatozoa after ipconazole exposure at 5, 10, 50 and 100 µM. Ipconazole led to a significant decrease in cell viability and a significant increase in ROS generation, as well as several changes in catalase and SOD activity. It also altered the molecular expression of structural and oxidative stress biomarkers in spermatozoa of both species.

### 3.1. Cell Viability

**Figure 1:**
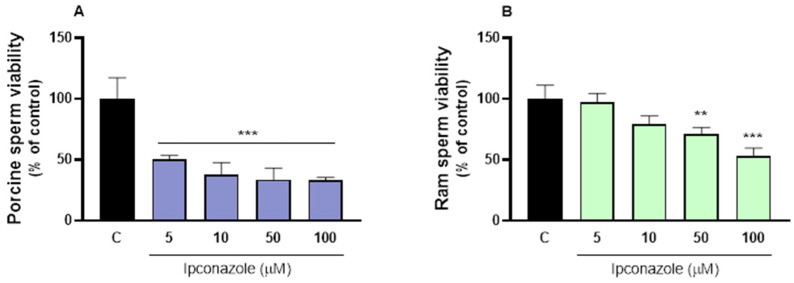
Cell viability assay in porcine ((**A**), left) and ram ((**B**), right) spermatozoa after 2 h exposure to ipconazole at concentrations of 5, 10, 50 and 100 µM. Data represent the mean ± standard error of three replicates per group, and data were analyzed using a one-way ANOVA. ** *p* ≤ 0.01 and *** *p* ≤ 0.001 represent significant differences compared to the control group (black bar).

The cell viability results evidenced the cytotoxic effect of ipconazole, as porcine sperm viability was significantly reduced (*p* ≤ 0.001) at all tested concentrations of ipconazole: 5 µM (50%), 10 µM (63%), 50 µM (66%) and 100 µM (67%) (Figure 1A). In addition, in ram sperm, ipconazole significantly reduced viability at concentrations of 50 µM (30%, *p* ≤ 0.01) and 100 µM (48%, *p* ≤ 0.001) (Supplementary Figure S1).

### 3.2. ROS Assay

**Figure 2:**
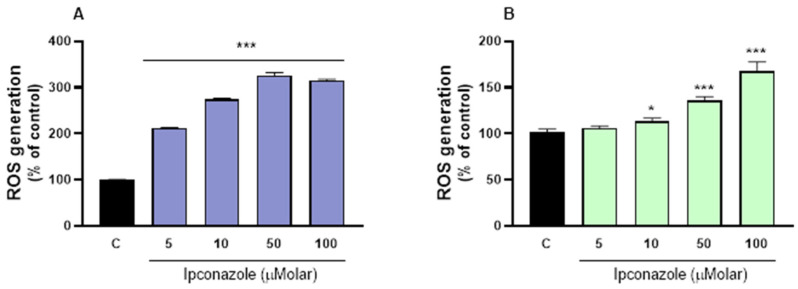
ROS generation assay in porcine ((**A**), left) and ram ((**B**), right) spermatozoa following exposure to 5, 10, 50 and 100 µM concentrations of the fungicide ipconazole for 2 h. Data represent the mean ± standard error of three replicates in each group, and data were analyzed by a one-way ANOVA. * *p* ≤ 0.05 and *** *p* ≤ 0.001 represent significant differences with respect to the control group (black bar).

Spermatozoa cells have a high metabolic rate and are very sensitive to oxidative stress. In this study, increased oxidative reactivity (ROS generation) was observed in porcine spermatozoa exposed to ipconazole concentrations of 5 µM (2-fold, compared to the control), 10 µM (2.7-fold), 50 µM (3-fold) and 100 µM (3-fold) (Figure 2A). In ram spermatozoa, ROS levels significantly increased after ipconazole exposure at 10 µM (1.13-fold, *p* ≤ 0.05), 50 µM (1.35-fold, *p* ≤ 0.01) and 100 µM (1.7-fold, *p* ≤ 0.001) ipconazole (Figure 2B).

### 3.3. Catalase Activity

**Figure 3:**
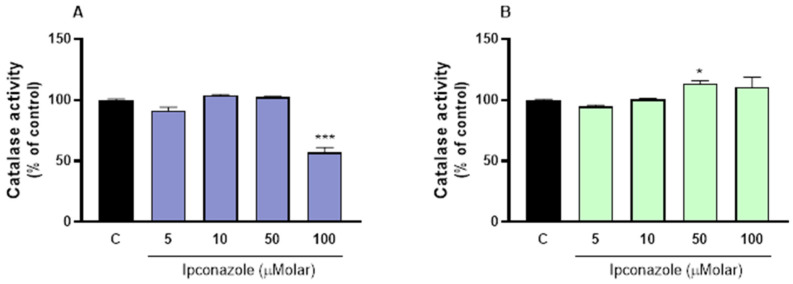
Catalase activity assay in porcine ((**A**), left) and ram ((**B**), right) spermatozoa following exposure to 5, 10, 50 and 100 µM concentrations of the fungicide ipconazole for 2 h. Data represent the mean ± standard error of three replicates in each group, and data were analyzed by a one-way ANOVA. * *p* ≤ 0.05 and *** *p* ≤ 0.001 represent significant differences with respect to the control group (black bar).

The antioxidant enzyme catalase catalyzes the decomposition of hydrogen peroxide (H_2_O_2_) into oxygen (O_2_) and water (H_2_O). In this study, we observed a significant (*p* ≤ 0.001) reduction (44%) in catalase activity in porcine spermatozoa after exposure to the higher concentration of ipconazole (100 µM) compared to the control (Figure 3A). In ram spermatozoa, however, catalase activity significantly (*p* ≤ 0.05) increased (13%) only at 50 µM compared to the control (Figure 3B).

### 3.4. SOD Activity

**Figure 4:**
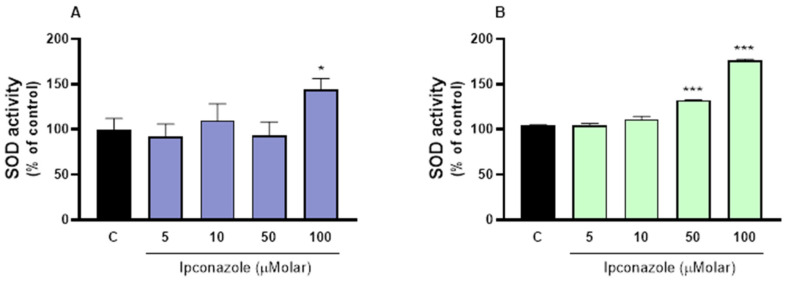
SOD activity assay in porcine ((**A**), left) and ram ((**B**), right) spermatozoa following exposure to 5, 10, 50 and 100 µM concentrations of the fungicide ipconazole for 2 h. Data represent the mean ± standard error of three replicates in each group, and data were analyzed by a one-way ANOVA. * *p* ≤ 0.05 and *** *p* ≤ 0.001 represent significant differences with respect to the control group (black bar).

SOD catalyzes the dismutation of superoxide into oxygen and hydrogen peroxide [2O_2_-(O_2_· + O_2_·) + 2H+ → H_2_O_2_ + O_2_]. Ipconazole-induced oxidative stress led to increased antioxidant defenses in both porcine and ram spermatozoa. Ipconazole at 100 µM induced a significant (*p* ≤ 0.05) increase (44%) in SOD activity in porcine spermatozoa (Figure 4A), and in ram spermatozoa, this increase occurred at concentrations of 50 (32%) and 100 (76%) µM ipconazole (Figure 4B).

### 3.5. Gene Expression by qPCR

**Figure 5:**
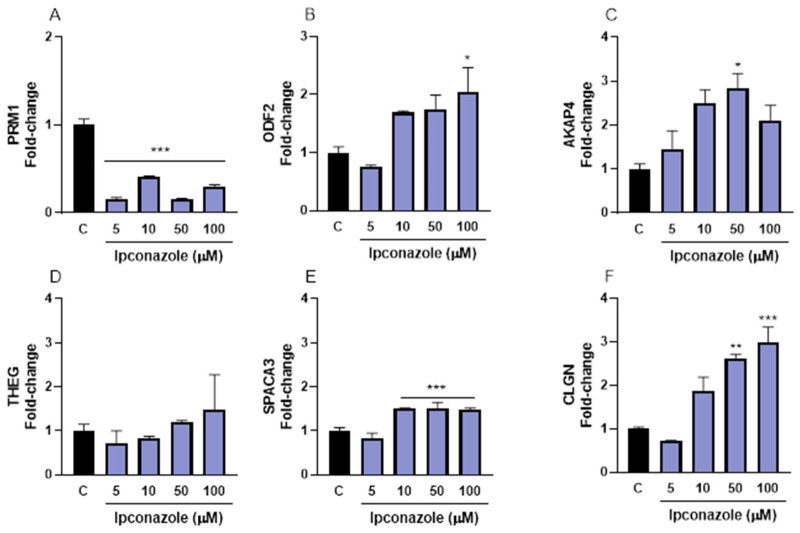
Gene expression of cellular structure (PRM1, (**A**); ODF2, (**B**); AKAP4, (**C**); THEG, (**D**); SPACA3, (**E**); and CLGN, (**F**)) biomarkers in porcine spermatozoa following exposure to 5, 10, 50 and 100 µM concentrations of the fungicide ipconazole for 2 h. Data represent the mean ± standard error of three replicates in each group, and data were analyzed by a one-way ANOVA. * *p* ≤ 0.05, ** *p* ≤ 0.01 and *** *p* ≤ 0.001 represent significant differences with respect to the control group (black bar).

We also evaluated the effect of ipconazole on the expression of structural biomarkers (PRM1, ODF2, AKAP4, THEG, SPACA3 and CLGN) of porcine spermatozoa. PRM1 mRNA expression was significantly (*p* ≤ 0.001) reduced at all doses of ipconazole (5 µM, 85%; 10 µM, 59%; 50 µM, 85%; and 100 µM, 71%) compared to the control (Figure 5A). Conversely, a significant increase (*p* ≤ 0.05) of 105% in ODF2 expression was observed at 100 µM ipconazole compared to the control (Figure 5B). AKAP4 expression was significantly (*p* ≤ 0.05) increased (183%) at 50 µM (Figure 5C). SPACA3 expression showed a 50% approximate increase (*p* ≤ 0.001) at 10, 50 and 100 µM (Figure 5E). Ipconazole significantly increased CLGN expression at 50 (160%, *p* ≤ 0.01) and 100 µM (200%, *p* ≤ 0.001) (Figure 5F).

**Figure 6:**
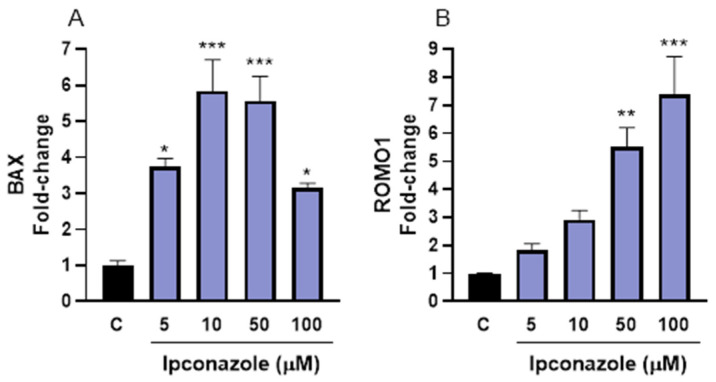
Gene expression of cell death (BAX, (**A**)) and oxidative stress (ROMO1, (**B**)) biomarkers in porcine spermatozoa following exposure to 5, 10, 50 and 100 µM concentrations of the fungicide ipconazole for 2 h. Data represent the mean ± standard error of three replicates in each group, and data were analyzed by a one-way ANOVA. * *p* ≤ 0.05, ** *p* ≤ 0.01 and *** *p* ≤ 0.001 represent significant differences with respect to the control group (black bar).

The oxidative effects of ipconazole may also modify the expression of molecular biomarkers related to cell death and oxidative stress. In porcine spermatozoa, ipconazole significantly elevated BAX expression at 5 µM (273%, *p* ≤ 0.05), 10 µM (480%, *p* ≤ 0.001), 50 µM (450%, *p* ≤ 0.001) and 100 µM (210%, *p* ≤ 0.05) (Figure 6A). Likewise, in porcine spermatozoa, the oxidative stress biomarker ROMO1 significantly increased when exposed to ipconazole at 50 µM (450%, *p* ≤ 0.001) and 100 µM (630%, *p* ≤ 0.001) (Figure 6B).

**Figure 7:**
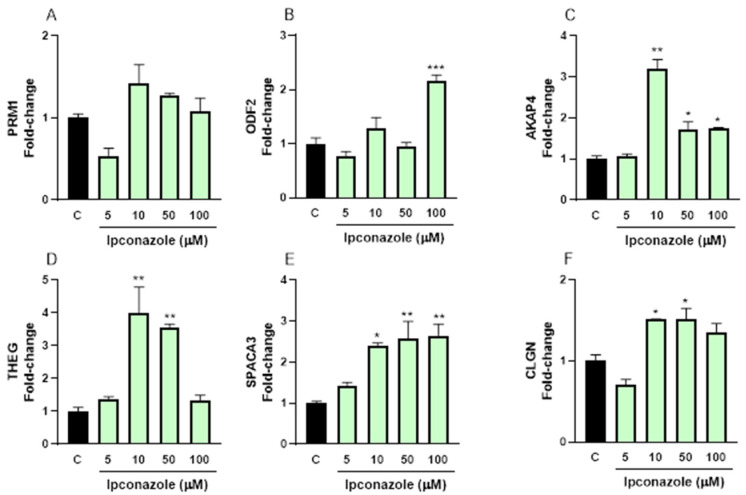
Gene expression of cellular structure (PRM1, (**A**); ODF2, (**B**); AKAP4, (**C**); THEG, (**D**); SPACA3, (**E**); and CLGN, (**F**)) biomarkers in ram spermatozoa following exposure to 5, 10, 50 and 100 µM concentrations of the fungicide ipconazole for 2 h. Data represent the mean ± standard error of three replicates in each group, and data were analyzed by a one-way ANOVA. * *p* ≤ 0.05, ** *p* ≤ 0.01 and *** *p* ≤ 0.001 represent significant differences with respect to the control group (black bar).

Our results reveal that ipconazole at 100 µM significantly increased (115%, *p* ≤ 0.001) ODF2 expression (Figure 7B). Ipconazole significantly upregulated AKAP4 expression at 10 µM (210%, *p* ≤ 0.01), 50 µM (71%, *p* ≤ 0.05) and 100 µM (73%, *p* ≤ 0.05) (Figure 7C). Additionally, ipconazole increased THEG expression at 10 µM (290%, *p* ≤ 0.01) and 50 µM (250%, *p* ≤ 0.01) (Figure 7D). Similarly, SPACA3 expression was significantly increased at 10 µM (140%, *p* ≤ 0.05), 50 µM (150%, *p* ≤ 0.01) and 100 µM (160%, *p* ≤ 0.01) (Figure 7E). Lastly, CLGN expression increased by 50% (*p* ≤ 0.05) after exposure to 10 and 50 µM ipconazole.

**Figure 8:**
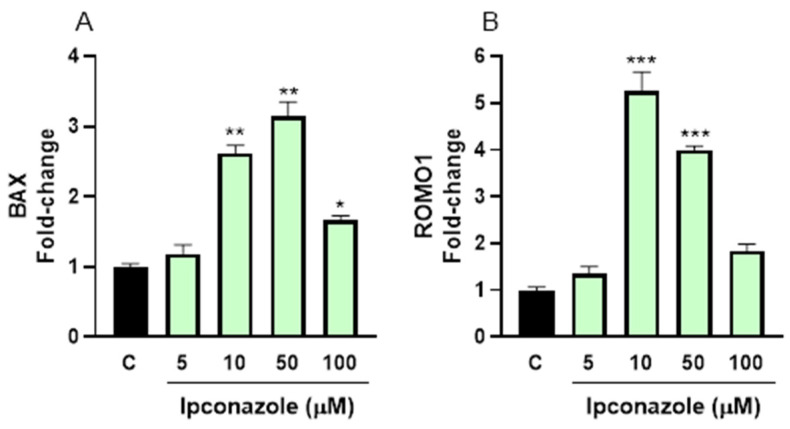
Gene expression of cell death (BAX, (**A**)) and oxidative stress (ROMO1, (**B**)) biomarkers in ram spermatozoa following exposure to 5, 10, 50 and 100 µM concentrations of the fungicide ipconazole for 2 h. Data represent the mean ± standard error of three replicates in each group, and data were analyzed by a one-way ANOVA. * *p* ≤ 0.05, ** *p* ≤ 0.01 and *** *p* ≤ 0.001 represent significant differences with respect to the control group (black bar).

Ipconazole significantly increased the cell death biomarker BAX by 160% at 10 µM (*p* ≤ 0.01), 210%at 50 µM (*p* ≤ 0.01) and 66% at 100 µM (*p* ≤ 0.05) (Figure 8A). Moreover, ipconazole markedly increased the oxidative stress biomarker ROMO1 at 10 µM (420%, *p* ≤ 0.001) and 50 µM (300%, *p* ≤ 0.001) (Figure 8B).

## 4. Discussion

Agricultural triazoles are used against pathogenic fungi on a variety of crops and are widely used worldwide. However, the inappropriate use or abuse of these fungicides causes physiological alterations in non-target organisms, such as humans and animals [19,20]. Negative consequences of exposure to triazoles (such as ipconazole) could include alteration of male reproductive health, and the use of animal models could be very practical in assessing fungicide toxicity on sperm physiology. In the present study, the fungicide ipconazole significantly reduced the viability of porcine and ram sperm, with porcine sperm being the most susceptible. These effects on sperm were also observed when the fungicide difenoconazole was administered to rats, where sperm numbers were reduced in all difeconazole-treated groups [21]. In an in vitro study in chickens, the triazoles epoxiconazole, tetraconazole, tebuconazole, difenoconazole, cyproconazole and metconazole were found to decrease sperm motility and velocity, and increase the percentage of sperm with an abnormal morphology [14].

Ipconazole-induced oxidative stress has been demonstrated experimentally in vitro and in vivo [16]. Data from this study suggest that ipconazole is able to alter the oxidative state by inducing ROS production and reducing or increasing the activity of the antioxidant enzymes SOD and catalase in porcine and ram spermatozoa. Overall, the oxidative effect (ROS generation) of ipconazole was higher in porcine spermatozoa than in ram spermatozoa, even reaching up to 3 times higher ROS levels compared to the control group. Several studies support our results, as pesticides such as prothioconazole, epoxiconazole, tetraconazole, tebuconazole, difenoconazole, cyproconazole and metconazole at different concentrations significantly increased ROS production in chicken spermatozoa [14]; or the additive mixture of tebuconazole and econazole induced ROS-dependent cytotoxicity in TM4 Sertoli cells [22].

The contrasting catalase responses (decreased in porcine sperm and increased in ram sperm) could be attributed to several factors. In this context, porcine sperm may be more sensitive to reactive oxygen species (ROS), leading to enzyme inactivation at higher ipconazole concentrations. In contrast, ram sperm might rely more heavily on catalase for peroxide detoxification, whereas porcine sperm may utilize alternative antioxidant systems, such as glutathione peroxidase. Additionally, the genes regulating catalase expression and activity may respond differently to oxidative stress in each species, resulting in distinct enzymatic responses [23]. The unexpected increase in superoxide dismutase (SOD) activity in both species could be explained by several mechanisms. As a primary antioxidant enzyme, SOD may be upregulated to counteract the increased superoxide production induced by ipconazole exposure. Furthermore, low-to-moderate oxidative stress can trigger adaptive cellular responses, including enhanced antioxidant enzyme activity. Additionally, oxidative stress may activate existing SOD molecules through post-translational modifications, increasing their activity without necessarily raising enzyme levels [24,25]. These species-specific responses suggest that porcine and ram spermatozoa have evolved different strategies to maintain a redox balance. Firstly, porcine sperm may rely more on SOD and alternative peroxide-scavenging systems, possibly due to their unique reproductive physiology or environmental exposures. Secondly, ram sperm appear to exhibit a more robust catalase response, which may reflect adaptations to their specific reproductive strategies or environmental challenges. Further research is needed to elucidate the molecular mechanisms underlying these differential responses, particularly the regulatory pathways governing antioxidant enzyme expression and activity in sperm cells. Investigating additional antioxidant systems and their interactions will provide a deeper understanding of species-specific adaptations and improve strategies for mitigating oxidative stress in reproductive toxicology.

The removal or reduction of intracellular ROS production is efficiently performed by antioxidant enzymes such as catalase and SOD. In our study, catalase enzyme activity in the porcine semen was significantly reduced at the highest dose of ipconazole, whereas in ram spermatozoa, catalase activity was significantly increased. However, we observed that SOD activity in both porcine and ram spermatozoa increased significantly at the highest doses of ipconazole. At the cellular level, pesticide-induced stress has been found to produce oxidative stress, which contributes to toxicity in the form of ROS such as hydrogen peroxide, superoxide and hydroxyl radical [26]. Under homeostasis conditions, ROS production in cells is very low, whereas stress conditions such as pesticide exposure elevate ROS levels in cells. ROS molecules are highly toxic and can oxidize most lipids, proteins and nucleic acids, subsequently leading to cell death due to lipid peroxidation, membrane damage and enzyme inactivation. Oxidative stress can also occur as damage to biological systems or by impairing antioxidant defense systems [27]. To cope with oxidative stress, cells use a complex antioxidant defense system, consisting of antioxidant enzymes such as SOD (catalyzes the dismutation of superoxide into molecular oxygen and hydrogen peroxide), catalase (H2O2 scavenging), and others that scavenge free radicals and peroxides [16,28].

In the present study, the effect of the fungicide ipconazole on the mRNA molecular expression of structural proteins (PRM1, ODF2, AKAP4, THEG, SPACA3 and CLGN) of porcine and ram spermatozoa was evaluated. The molecular expression of PRM1 (sperm-specific protein essential for fertilization) was significantly reduced only in porcine spermatozoa, which could indicate that ipconazole alters the action of PRM1 and may affect sperm parameters and impair sperm shape or motility, ultimately leading to infertility [29,30,31]. When the molecular expression of ODF2 was assessed by ipconazole, its levels were found to be significantly increased in both species. ODF2 is a microtubule-associated coiled-coil protein in the tail of mammalian spermatozoa [32,33,34], and is a component of the centrosomal scaffold [35]. The disruption of ODF2 leads to preimplantation lethality, suggesting that ODF2 is crucial for cilia formation at the initial attachment of centrioles to membranes [36].

The AKAP4 protein has been identified in the sperm flagellum of several species and has been studied mainly for its role in sperm motility [37,38]. In this study, the fungicide ipconazole produced a significant increase in the molecular expression of AKAP4 mRNA in porcine and ram spermatozoa. In contrast, a study with mouse spermatozoa exposed to the pesticide bifenthrin showed a decrease in AKAP4 protein expression, and the same effect was observed with the use of fipronil sulphone in boar spermatozoa [39].

The molecular expression of mRNAs of other proteins involved in the fertilization capacity of spermatozoa, such as THEG, SPACA3 and CLGN, was also evaluated in this study. After the administration of ipconazole to porcine and ram spermatozoa, the molecular expression of these mRNAs was altered, especially at higher ipconazole concentrations. At present, there are no reports on the effect of chemical compounds on these proteins, so in this section we have decided to provide a descriptive discussion of the functionality of these proteins. The molecular expression of THEG in sperm suggests that THEG may play an important role in the successful differentiation of male germ cells [40]. The SPACA3 gene encodes sperm lysozyme-like protein 1 that plays a vital role in sperm-ocyte interaction and fertilization, and a connection between SPACA3 expression and human sperm acrosome integrity and fertility can be suggested [41]. CLGN is a testis-specific molecular chaperone that is required for α/β-fertilin heterodimerization and the appearance of β-fertilin on the sperm surface, and during spermatogenesis, it acts as a chaperone for a variety of proteins that are important for sperm adhesion to the zona pellucida of the egg and for subsequent penetration into the zona pellucida, and is necessary for normal sperm migration from the uterus to the oviduct [42].

Finally, the molecular expression of a biomarker of cell death (BAX) and a biomarker of oxidative stress (ROMO1) was evaluated in this study. Both biomarkers were overexpressed by higher concentrations of ipconazole in porcine and ram spermatozoa. ROMO1 is a mitochondrial membrane protein that regulates mitochondrial ROS production within cells, especially in cells with elevated metabolism, such as spermatozoa [43], and is involved in cellular processes, such as proliferation, senescence and cell death by inducing BAX expression [44]. Another study reports that ROMO1 plays a critical role in embryonic development by regulating mitochondrial morphology, function and apoptosis in swine [43]. The positive association between ROMO1 and BAX has been demonstrated in another study [45] in canine spermatozoa, where a decrease in ROMO1 resulted in a reduction in BAX expression. Also, many studies indicate that spermatozoa exposure to pesticides induces the expression of oxidative stress and cell death such as BAX [46,47,48].

## 5. Conclusions

To our knowledge, this is the first study to evaluate the cytotoxic effect of the triazole ipconazole on mammalian spermatozoa (porcine and ram). The spermiotoxic effect of ipconazole reduced cell viability and produced oxidative stress, which would be the likely cause of the molecular alteration of the gene expression of fertility-related structural proteins in male mammals. However, further studies in other animal species are needed to establish the mechanistic effect of triazole pesticides on fertility in animals.

## Data Availability

The data that support the findings of this study are available from the corresponding author upon reasonable request.

## Supplementary Materials

The following are available online at https://www.mdpi.com/article/10.3390/toxics13030176/s1, Figure S1: Structural alterations in spermatozoa of pigs and rams exposed to ipconazole.

## References

[1] Mehrpour O., Karrari P., Zamani N., Tsatsaki A.M., Abdollahi M. (2014). Occupational exposure to pesticides and consequences on male semen and fertility: A review. Toxicol. Lett..

[2] Bi R., Ou M., Zhou S., Geng S., Zheng Y., Chen J., Mo R., Li Y., Xiao G., Chen X. (2023). Degradation strategies of pesticide residue: From chemicals to synthetic biology. Synth. Syst. Biotechnol..

[3] Ahmad M.F., Ahmad F.A., Alsayegh A.A., Zeyaullah M., AlShahrani A.M., Muzammil K., Saati A.A., Wahab S., Elbendary E.Y., Kambal N. (2024). Pesticides impacts on human health and the environment with their mechanisms of action and possible countermeasures. Heliyon.

[4] Hu R., Huang X., Huang J., Li Y., Zhang C., Yin Y., Chen Z., Jin Y., Cai J., Cui F. (2015). Long- and short-term health effects of pesticide exposure: A cohort study from China. PLoS ONE.

[5] Moreira S., Pereira S.C., Seco-Rovira V., Oliveira P.F., Alves M.G., Pereira M.L. (2021). Pesticides and male fertility: A dangerous crosstalk. Metabolites.

[6] Giulioni C., Maurizi V., Castellani D., Scarcella S., Skrami E., Balercia G., Galosi A.B. (2022). The environmental and occupational influence of pesticides on male fertility: A systematic review of human studies. Andrology.

[7] Xia Y., Han Y., Wu B., Wang S., Gu A., Lu N. (2008). The relation between urinary metabolite of pyrethroid insecticides and semen quality in humans. Fertil Steril..

[8] Melgarejo M., Mendiola J., Koch H.M., Moñino-García M., Noguera-Velasco J.A., Torres-Cantero A.M. (2015). Associations between urinary organophosphate pesticide metabolite levels and reproductive parameters in men from an infertility clinic. Environ. Res..

[9] Vitku J., Heracek J., Sosvorova L., Hampl R., Chlupacova T., Hill M., Sobotka V., Bicikova M., Starka L. (2016). Associations of bisphenol A and polychlorinated biphenyls with spermatogenesis and steroidogenesis in two biological fluids from men attending an infertility clinic. Environ. Int..

[10] Wang Y.-X., You L., Zeng Q., Sun Y., Huang Y.-H., Wang C., Wang P., Cao W.-C., Yang P., Li Y.-F. (2015). Phthalate exposure and human semen quality: Results from an infertility clinic in China. Environ. Res..

[11] Jurewicz J., Radwan M., Sobala W., Ligocka D., Radwan P., Bochenek M., Hawula W., Jakubowski L., Hanke W. (2013). Human urinary phthalate metabolites level and main semen parameters, sperm chromatin structure, sperm aneuploidy and reproductive hormones. Reprod. Toxicol..

[12] Jia M., Teng M., Tian S., Yan J., Meng Z., Yan S., Li R., Zhou Z., Zhu W. (2021). Effects of penconazole enantiomers exposure on hormonal disruption in zebrafish Danio rerio (Hamilton, 1822). Environ. Sci. Pollut. Res. Int..

[13] Lee W.Y., Lee R., Park H.J. (2024). Tebuconazole Induces Mouse Fetal Testes Damage Via ROS Generation in an Organ Culture Method. Int. J. Mol. Sci..

[14] Serra L., Bourdon G., Estienne A., Fréville M., Ramé C., Chevaleyre C., Didier P., Chahnamian M., Ganier P., Pinault F. (2023). Triazole pesticides exposure impaired steroidogenesis associated to an increase in AHR and CAR expression in testis and altered sperm parameters in chicken. Toxicol. Rep..

[15] World Health Organization (2021). WHO Laboratory Manual for the Examination and Processing of Human Semen.

[16] Villaorduña C., Barrios-Arpi L., Lira-Mejía B., Ramos-Gonzalez M., Ramos-Coaguila O., Inostroza-Ruiz L., Romero A., Rodríguez J.-L. (2024). The Fungicide Ipconazole Can Activate Mediators of Cellular Damage in Rat Brain Regions. Toxics.

[17] Barrios-Arpi L., Arias Y., Lopez-Torres B., Ramos-Gonzalez M., Ticli G., Prosperi E., Rodríguez J.-L. (2022). In Vitro Neurotoxicity of Flumethrin Pyrethroid on SH-SY5Y Neuroblastoma Cells: Apoptosis Associated with Oxidative Stress. Toxics.

[18] Weydert C.J., Cullen J.J. (2010). Measurement of superoxide dismutase, catalase and glutathione peroxidase in cultured cells and tissue. Nat. Protoc..

[19] Liu N., Jin X., Zhou J., Wang Y., Yang Q., Wu F., Giesy J.P., Johnson A.C. (2018). Predicted no-effect concentration (PNEC) and assessment of risk for the fungicide, triadimefon based on reproductive fitness of aquatic organisms. Chemosphere.

[20] Lv X., Pan L., Wang J., Lu L., Yan W., Zhu Y., Xu Y., Guo M., Zhuang S. (2017). Effects of triazole fungicides on androgenic disruption and CYP3A4 enzyme activity. Environ. Pollut..

[21] Pereira V.R., Pereira D.R., de Melo Tavares Vieira K.C., Ribas V.P., Constantino C.J.L., Antunes P.A., Favareto A.P.A. (2019). Sperm quality of rats exposed to difenoconazole using classical parameters and surface-enhanced Raman scattering: Classification performance by machine learning methods. Environ. Sci. Pollut. Res. Int..

[22] Petricca S., Carnicelli V., Luzi C., Cinque B., Celenza G., Iorio R. (2023). Oxidative Stress, Cytotoxic and Inflammatory Effects of Azoles Combinatorial Mixtures in Sertoli TM4 Cells. Antioxidants.

[23] Zamocky M., Furtmüller P.G., Obinger C. (2008). Evolution of catalases from bacteria to humans. Antioxid. Redox Signal..

[24] Wang Y., Branicky R., Noë A., Hekimi S. (2018). Superoxide dismutases: Dual roles in controlling ROS damage and regulating ROS signaling. J. Cell Biol..

[25] Van Raamsdonk J.M., Hekimi S. (2012). Superoxide dismutase is dispensable for normal animal lifespan. Proc. Natl. Acad. Sci. USA.

[26] D’Autréaux B., Toledano M.B. (2007). ROS as signaling molecules: Mechanisms that generate specificity in ROS homeostasis. Nat. Rev. Mol. Cell. Biol..

[27] Ighodaro O.M., Akinloye O.A. (2018). First line defence antioxidants-superoxide dismutase (SOD), catalase (CAT) and glutathione peroxidase (GPX): Their fundamental role in the entire antioxidant defence grid. Alex. J. Med..

[28] Jomova K., Alomar S.Y., Alwasel S.H., Nepovimova E., Kuca K., Valko M. (2024). Several lines of antioxidant defense against oxidative stress: Antioxidant enzymes, nanomaterials with multiple enzyme-mimicking activities, and low-molecular-weight antioxidants. Arch. Toxicol..

[29] Pardede B.P., Agil M., Supriatna I. (2020). Protamina y otras proteínas en el esperma y el plasma seminal como marcadores moleculares de la fertilidad del toro. Vet. World.

[30] Oliva R. (2006). Protaminas e infertilidad masculina. Hum. Reprod. Update.

[31] Jodar M., Oliva R. (2014). Alteraciones de la protamina en espermatozoides humanos. Adv. Exp. Med. Biol..

[32] Nakagawa Y., Yamane Y., Okanoue S., Tsukita S., Tsukita S. (2001). Outer dense fiber 2 is a widespread centrosome scaffold component preferentially associated with mother centrioles: Its identification from isolated centrosomes. Mol. Biol. Cell.

[33] Brohmann H., Pinnecke S., Hoyer-Fender S. (1997). Identification and characterization of new cDNAs encoding outer dense fiber proteins of rat sperm. J. Biol. Chem..

[34] Shao X., Tarnasky H.A., Schalles U., Oko R., van der Hoorn F.A. (1997). Interactional cloning of the 84-kDa major outer dense fiber protein Odf84. Leucine zippers mediate associations of Odf84 and Odf27. J. Biol. Chem..

[35] Schalles U., Shao X., van der Hoorn F.A., Oko R. (1998). Developmental expression of the 84 kDa ODF sperm protein: Localization to both the cortex and medulla of outer dense fibers and to the connecting piece. Dev. Biol..

[36] Salmon N.A., Reijo Pera R.A., Xu E.Y. (2006). A gene trap knockout of the abundant sperm tail protein, outer dense fiber 2, results in preimplantation lethality. Genesis.

[37] Carrera A., Moos J., Ning X.P., Gerton G.L., Tesarik J., Kopf G.S., Moss S.B. (1996). Regulation of protein tyrosine phosphorylation in human sperm by a calcium/calmodulin-dependent mechanism: Identification of A kinase anchor proteins as major substrates for tyrosine phosphorylation. Dev. Biol..

[38] Vijayaraghavan S., Stephens D.T., Trautman K., Smith G.D., Khatra B., da Cruz e Silva E.F., Greengard P. (1996). Sperm motility development in the epididymis is associated with decreased glycogen synthase kinase-3 and protein phosphatase 1 activity. Biol. Reprod..

[39] Adikari A.A.D.I., Yi Y.-J. (2023). The pesticide metabolite fipronil sulfone diminishes the fertilizing capacity of boar spermatozoa. Korean J. Agric. Sci..

[40] Mannan A.U., Nayernia K., Mueller C., Burfeind P., Adham I.M., Engel W. (2003). Male mice lacking the Theg (testicular haploid expressed gene) protein undergo normal spermatogenesis and are fertile. Biol. Reprod..

[41] Saadat Maryan H., Ghasemian F., Bahadori M.H. (2023). Effects of cryopreservation in the presence of Natural Deep Eutectic Solvents (NADESs) on sperm parameters. Cryobiology.

[42] Yamagata K., Nakanishi T., Ikawa M., Yamaguchi R., Moss S.B., Okabe M. (2002). Sperm from the calmegin-deficient mouse have normal abilities for binding and fusion to the egg plasma membrane. Dev. Biol..

[43] Zhou D., Sun M.-H., Lee S.-H., Cui X.-S. (2021). ROMO1 is required for mitochondrial metabolism during preimplantation embryo development in pigs. Cell Div..

[44] Jo M.J., Kim B.G., Park S.H., Kim H.J., Jeong S., Kim B.R., Kim J.L., Na Y.J., Jeong Y.A., Yun H.K. (2020). Romo1 Inhibition Induces TRAIL-Mediated Apoptosis in Colorectal Cancer. Cancers.

[45] Qamar A.Y., Fang X., Bang S., Kim M.J., Cho J. (2020). Effects of kinetin supplementation on the post-thaw motility, viability, and structural integrity of dog sperm. Cryobiology.

[46] Yang X., Yu X., Sun N., Shi X., Niu C., Shi A., Cheng Y. (2022). Glyphosate-based herbicide causes spermatogenesis disorder and spermatozoa damage of the Chinese mitten crab (*Eriocheir sinensis*) by affecting testes characteristic enzymes, antioxidant capacities and inducing apoptosis. Toxicol. Appl. Pharmacol..

[47] Wang Z., Li R., Yan Z., Fu H., Zhu Y. (2020). Injury of procymidone on testis and sperm in the male adolescent mice. Wei Sheng Yan Jiu..

[48] Pham T.H., Derian L., Kervarrec C., Kernanec P.Y., Jégou B., Smagulova F., Gely-Pernot A. (2019). Perinatal Exposure to Glyphosate and a Glyphosate-Based Herbicide Affect Spermatogenesis in Mice. Toxicol. Sci..

