# Supplementary material for: Oxidative and Molecular–Structural Alterations of Spermatozoa in Swine and Ram Exposed to the Triazole Ipconazole
Source: Toxics. 2025 Feb 28;13(3):176. doi: 10.3390/toxics13030176 (PMC11945538; doi:10.3390/toxics13030176)
Supplement: Supplementary file 1 [file toxics-13-00176-s001.zip › toxics-3463140-supplementary.pdf]

Figure S1. Structural alterations in spermatozoa of pigs and rams exposed to ipconazole

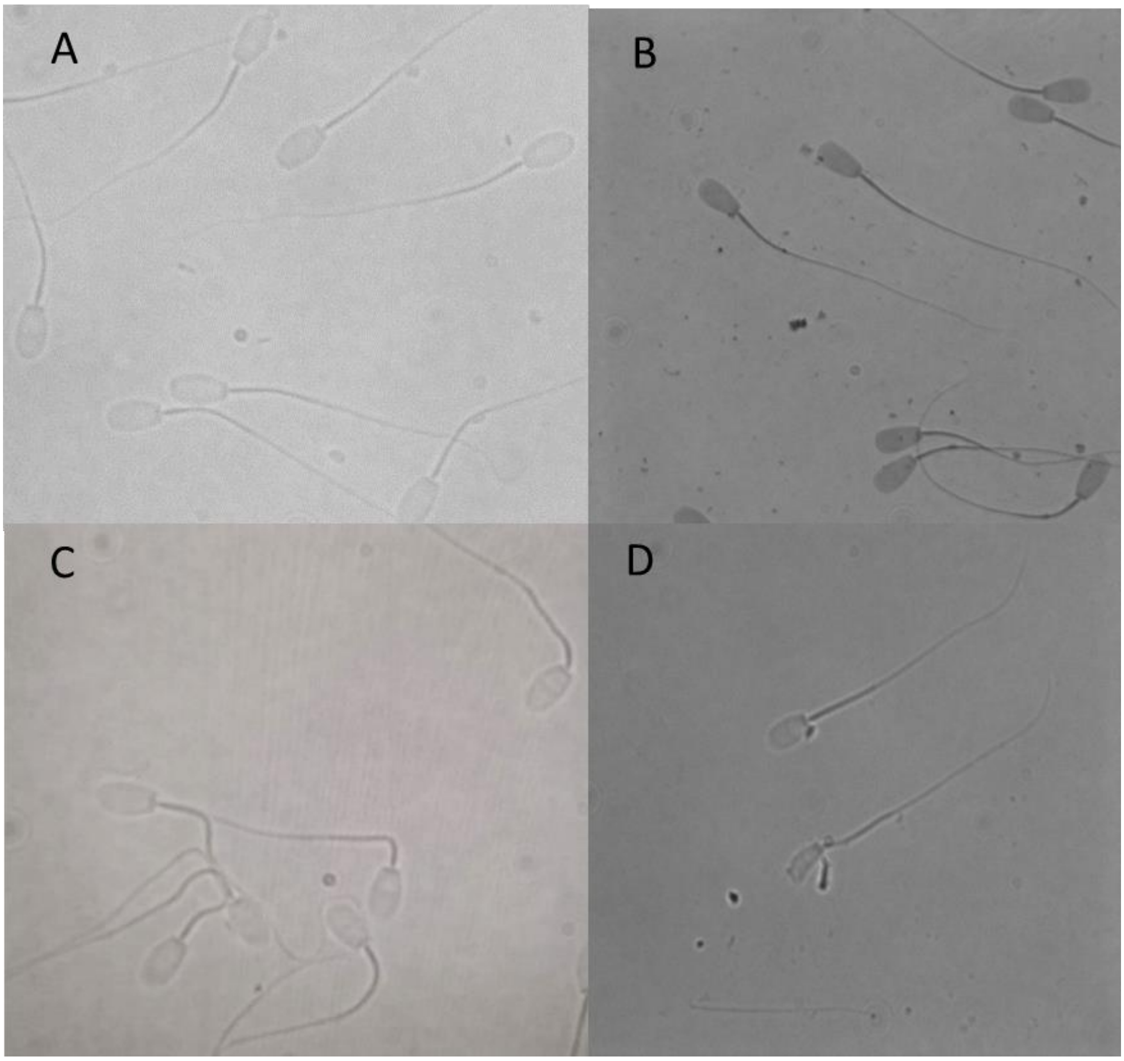

Figure. Spermatozoa cells in ram (A, left) and porcine (B, right), control without ipconazole exposure. Spermatozoa after 2-hour exposure to ipconazole at concentrations of 100 μM in ram (C, left) and spermatozoa after 2-hour exposure to ipconazole at concentrations of 100 μM in porcine (D, right). The image is at 1000X in phase contrast microscope: Spermatozoa in the upper image (A,B) show viability in their spermatic membrane as shown in the image, the tail and intermediate piece do not show morphological alteration. The lower image shows spermatozoa with morphological alteration in the tail and head which reduces their viability affecting sperm motility.
